# Deep learning‐based 3D classification of head and neck cancer PET/MRI: Radiologist comparison and Grad‐CAM interpretability

**DOI:** 10.1111/cpf.70030

**Published:** 2025-09-25

**Authors:** Joonas Liedes, Jussi Hirvonen, Oona Rainio, Sarita Murtojärvi, Simona Malaspina, Riku Klén, Jukka Kemppainen

**Affiliations:** ^1^ Turku PET Centre University of Turku and Turku University Hospital Turku Finland; ^2^ Department of Radiology University of Turku and Turku University Hospital Turku Finland; ^3^ Department of Otorhinolaryngology Turku University Hospital Turku Finland; ^4^ Department of Clinical Physiology and Nuclear Medicine Turku University Hospital Turku Finland

**Keywords:** deep learning, Grad‐CAM, head and neck cancer, interpretable AI, PET/MRI

## Abstract

**Purpose:**

To develop and evaluate a three‐dimensional convolutional neural network for automated classification of PET/MRI images in head and neck cancer (HNC) patients, assessing its performance against radiologist interpretation and its potential as a diagnostic aid.

**Methods:**

Data from 202 patients with HNC who underwent ^18^F‐FDG PET/MRI were used to train and validate PET‐, MRI‐, and PET/MRI‐based models. Of these data, 101 patients were labelled as positive in terms of having HNC, and 101 patients as negative. An additional test set of 20 patients was also evaluated, where 10 patients were labelled as positive and 10 as negative. The model performance was assessed using sensitivity, specificity, accuracy, and AUC. Grad‐CAM was utilised to improve interpretability and classification results on the test set were compared with a radiologist.

**Results:**

The PET‐based model achieved an AUC of 0.92 on the test set, with an accuracy of 90%, a sensitivity of 100% and a specificity of 80%. PET/MRI and MRI‐based models underperformed relative to the PET‐based model. The radiologist achieved perfect classification accuracy. Analysis of Grad‐CAM showed that the model classifications are based on real areas of interest. In addition, it gave valuable insight into using similar systems in identifying false positive findings.

**Conclusion:**

The PET‐based model demonstrated high sensitivity, indicating its potential as a pre‐screening tool for HNC. However, specificity requires improvement to reduce false‐positive rates. Enhanced datasets and refinement of model architecture will be crucial before clinical adoption. Grad‐CAM provides valuable insights into model decisions, aiding clinical integration.

## 1 INTRODUCTION

Head and neck cancer (HNC, incl. cancers originating from the oral cavity, larynx, nasopharynx, oropharynx, hypopharynx, and salivary glands) is common and poses a significant threat to public health, with nearly 900 000 new cases worldwide in 2020 (Cancer (IARC), n.d.). The majority of HNC originates from the squamous cells of the mucosa. However, other types of cancer, such as adenocarcinomas and adenocystic carcinomas, are regularly found in the head and neck region, as well as cancers of the salivary glands and the thyroid gland. The head and neck area is one of the body′s most crucial and complex anatomical structures, and diseases in this area can have a seriously adverse impact on the patient′s health and well‐being.

The intricate anatomy of the region makes the diagnosis challenging and time‐consuming. Early and accurate diagnosis is essential, as advanced and recurrent diseases have a poor prognosis (Specenier and Vermorken, 2008). Conventional anatomical imaging modalities, such as computed tomography (CT) and magnetic resonance imaging (MRI), often have limited ability to distinguish between benign changes, such as fibrosis and inflammation, and recurrent disease. Therefore, imaging is often supplemented with positron emission tomography (PET), a molecular imaging method. PET/CT with ^18^F‐fluorodeoxyglucose (^18^F‐FDG) has been established as an accurate method for recurrent disease detection with improved fidelity in separating fibrosis and inflammation from cancer compared to conventional imaging (Kao et al., 2009). Moreover, MRI has been proposed as an alternative to CT due to its superior soft tissue contrast and fewer artifacts from ferromagnetic foreign bodies, such as dental implants and reconstruction materials (such as screws and plates) (Loeffelbein et al., 2012).

However, interpretation of fusion imaging is difficult and time‐consuming and often suffers from interobserver variability and low reproducibility (Riegel et al., 2006). In recent years, deep learning has emerged as a capable method for automated image interpretation tasks. This approach has been successfully implemented in medical computer vision tasks (Ronneberger et al., 2015). Using computer vision for medical image segmentation has several benefits. Firstly, these algorithms can match or even surpass the accuracy of trained specialists (Esteva et al., 2017; Hwang et al., 2019). Secondly, they are reasonably fast to train, after which they yield predictions in seconds. Moreover, the predictions are more reproducible due to the lack of human reader intra‐ or interobserver variability. In this study, we aim to demonstrate these benefits using a three‐dimensional (3D) convolutional neural network (CNN) to automatically classify PET/MRI images of patients with cancer of the head and neck area. This study adds to our previous work on the subject, where we have shown the feasibility of using 2D CNN's for HNC classification from PET images (Hellström et al., 2023).

## 2 MATERIALS

### 2.1 Training data

Patients with HNC referred for ^18^F‐FDG PET/MRI scans in Turku University Hospital during 2014–2022 were included in the training data with the following criteria: previously diagnosed histologically confirmed malignant tumour of the head and neck region, and ^18^F‐FDG as the radiotracer used. A total of 202 consecutive patients were included, consisting of 101 patients with cancer and 101 patients without cancer, as detailed in our previous work (Hellström et al., 2023), with the exception of this study including an additional positive patient that had a SCC of the hypopharynx and a negative patient that had been treated for a SCC of the right tonsil. Ground truth classification was based on clinical readings combined with histological confirmation of disease or absence of disease at follow‐up. Equivocal cases were resolved by histological confirmation or clinical follow‐up, and assigned accordingly to the positive or negative group. The majority of the patients had received treatment with curative intention to the HNC before their PET/MRI scan. However, some primary scans in the event of an unknown primary were also included in the positive subgroup. In addition, the data contained more rare types of HNC, as detailed below (Table 1). The rationale behind this decision was to mimic the real‐world head and neck PET/MRI interpretation setting to evaluate the applicability of these systems to the broader clinical workflow. The patients in the negative subgroup had been previously treated successfully for HNC and were classified as negative if recurrence was not detected within 1 year of the initial scan. Only one scan was chosen per patient in the event of multiple scans within the observation period. In these cases, the scan closest to the time of diagnosis was chosen. The 50/50‐ratio of positive to negative samples was chosen to facilitate a more efficient training process. The different locations of HNC and cancer types included in the training data are detailed in Table 1. The gender and age characteristics of this study population are presented in Table 2.

**TABLE 1:** Tumour histologies included in the training data grouped by location.

Location	Histology	*N*
Pharynx	SCC	82
	Clear cell carcinoma	1
Oral cavity	SCC	47
	Adenocystic carcinoma	4
Larynx	SCC	20
Unknown primary	SCC	18
Nasal cavity	SCC	7
	Epithelial carcinoma	1
Salivary glands	SCC	2
	Asinus cell carcinoma	1
	Adenocarcinoma	1
	Mucoepidermoid carcinoma	1
	Epithelial carcinoma	1
	Salivary duct carcinoma	1
Oesophagus	SCC	2
	Adenocarcinoma	2
Sinuses	SCC	2
Skin	SCC	2
	Merkel cell carcinoma	1
	Adnexal carcinoma	1
	Angiosarcoma	1
Thyroid	Papillar carcinoma	2
	Follicular carcinoma	1
Skull base	Chondrosarcoma	1

**TABLE 2:** Gender and age characteristics of the training data. Age is displayed as mean ± SD.

Gender	*N*	Age	Median age
Male	134	63.6 ± 11.1	64.0
Female	68	61.7 ± 12.8	61.5
Total	202	63.0 ± 11.7	64.0

The ^18^F‐FDG PET/MRI scans were performed with 3T Philips Ingenuity TF PET/MR scanner (Philips Health Care) and 3T SIGNA™ PET/MR with QuantWorks (GE Healthcare). The PET images were cropped to the dimensions of the corresponding MRI sequence of the head and neck area, with the final data set consisting of 202 PET/MRI images with 32‐66 consecutive transaxial slices of 512×512 pixels. PET‐MRIs until 3/2020 were conducted using a sequential Ingenuity 3T TF PET‐MRI system (Philips Healthcare), with a SENSE neurovascular coil. These patients summed up to 74 scans out of the total 202. Of these scans, 30 were positive and 44 negative. For MRI acquisition with this scanner, transaxial sequences included T1, T1 SPIR and T2. The T1‐weighted sequences focused on the region of the primary tumour. Contrast agent was administered with the T1 SPIR scans. T2‐weighted sequences were utilised to provide precise anatomical delineation of both the tumour and adjacent lymph node regions. Of the 30 positive cases, 23 were T1 SPIR, 4 were T1, and 3 were T2. Of the 44 negative cases, 28 were T1 SPIR, 15 were T2, and 1 was T1. Sequences were chosen based on availability and image quality.

With the Philips Ingenuity TF PET/MR scanner PET imaging was initiated immediately following the MRI acquisition. The PET scan employed a transaxial field of view (FOV) of 576 mm. Image reconstruction was performed using the system's default “Blob‐OS‐TF” algorithm, a 3D ordered subset iterative time‐of‐flight (TOF) reconstruction method. The final PET images were reconstructed on a 144 × 144 matrix, yielding a voxel size of 4 × 4 × 4 mm³. Standard corrections required for quantitative PET imaging, including attenuation, randoms, scatter, dead‐time, decay, and detector normalisation, were incorporated. Attenuation correction was achieved using Dixon MRI‐based sequences, which spanned from the level of the forehead to the groin. The correction employed a 3‐segment model, which distinguished between air, lung tissue, and soft tissue.

After 3/2020 3T SIGNA™ PET/MR with QuantWorks (GE Healthcare) was introduced to our centre and subsequent scans were carried out using it. These scans formed the remaining 128 out of 202 training scans. Of these scans, 71 were positive and 57 negative. This scanner employed a transaxial FOV of 600 mm. Image reconstruction was performed using the system's Q.Clear algorithm. The final PET images were reconstructed on a 256 × 256 matrix, yielding a voxel size of 2.3 × 2.3 × 2.8 mm³. Standard corrections required for quantitative PET imaging, including attenuation, randoms, scatter, dead‐time, decay, and detector normalisation, were incorporated. MRI sequences utilised from this machine included T1 and T2‐weighted sequences. Of the 71 positive cases, 64 were T1 and 7 were T2. All 57 negative cases were T2. Sequences were chosen based on availability and image quality.

### 2.2 Test data

A sample of 20 consecutive patients with HNC referred for ^18^F‐FDG PET/MRI scans in Turku University Hospital during 2022–2023 was included in the independent test data with the same criteria as the training data. The data consisted of 10 patients with cancer and 10 patients without cancer. The following types of HNC were included in the test data: 9 SCCs of the pharynx, 4 SCCs of the larynx, 4 SCCs of the oral cavity, 1 clear cell carcinoma of the pharynx, 1 adenocarcinoma of the pharynx, 1 myoepithelial carcinoma of the sinuses and 1 unknown primary (SCC). One patient had an SCC of the oral cavity and pharynx. The different histologies and locations of the test data are presented in Table 3. The gender and age characteristics of the test population are presented in Table 4.

**TABLE 3:** Tumour histologies included in the test data grouped by location.

Location	Histology	*N*
Pharynx	SCC	9
	Clear cell carcinoma	1
	Adenocarcinoma	1
Larynx	SCC	4
Oral Cavity	SCC	3
Sinuses	Myoepithelial carcinoma	1
Unknown Primary	SCC	1

**TABLE 4:** Gender and age characteristics of the test data. Age is displayed as mean ± SD.

Gender	*N*	Age	Median age
Male	12	69.7 ± 6.3	71.5
Female	8	57.9 ± 10.6	55.0
Total	20	65.0 ± 10.1	66.5

The ^18^F‐FDG PET/MRI scans were performed with the same 3T SIGNA™ PET/MR with QuantWorks (GE Healthcare) as described in the previous chapter. The PET images were cropped to the dimensions of the corresponding MRI sequence of the head and neck area with the final data set consisting of 20 PET/MRI images with 48–50 consecutive transaxial slices of 512×512 pixels.

## 3 METHODS

### 3.1 Image analysis

To prepare the images for the deep learning model, they were co‐registered, and the PET images were resliced to the corresponding MRI′s voxel space using Carimas software (Rainio et al., 2023). PET/MRI reports written by nuclear medicine specialists and radiologists along with clinical information from the patient records were utilised in the analysis. Based on this information, binary labels were assigned to the patients.

### 3.2 Data pre‐processing

The following pre‐processing steps were conducted using Python. The images were first resized in‐plane from 512×512 pixels to 64×64 pixels to increase the batch size and reduce the computational overhead. Voxel size ranged from (0.4, 0.4, 3.4) to (0.7, 0.7, 4.5) in mm. The discrepancy along the x‐ and y‐axes is roughly two‐fold and likely to impact classification accuracy. Therefore, the voxel sizes were interpolated using the highest resolution of the data set. In addition, the image stacks were cropped along the *z*‐axis to a common size of 32 image slices. For the subgroup with cancer, the slices were chosen based on the tumour's location along the z‐axis by selecting the slices from both sides of the centremost mask slice. Importantly, this does not force the tumour to be centred within the window; rather, the lesion could appear at different positions along the depth axis, while surrounding normal tissue context was preserved. For the negative images, 32 consecutive slices were chosen randomly. This resulted in 202 PET/MRI images with dimensions of 64×64×32 pixels. Voxel values were then linearly scaled between 0 and 1 due to the differing pixel value ranges between PET and MRI. The scaling was applied per patient per 3D image utilising the minima and maxima acquired from the image. A simple augmentation pipeline incorporating flipping and random 90‐degree rotations was built to quadruple the size of the training set during the fivefold cross‐validation, resulting in 805 training samples for the first two training folds and 810 samples for the last three. Pre‐processing of the independent test set was done similarly, except no cropping was done along the *z*‐axis as a sliding window approach was used for inference. A sliding window enables models to handle varying input sizes and focus on specific areas. It is a technique where a fixed‐size window moves across an input volume to process regions sequentially.

### 3.3 Model architecture

**FIGURE 1:**
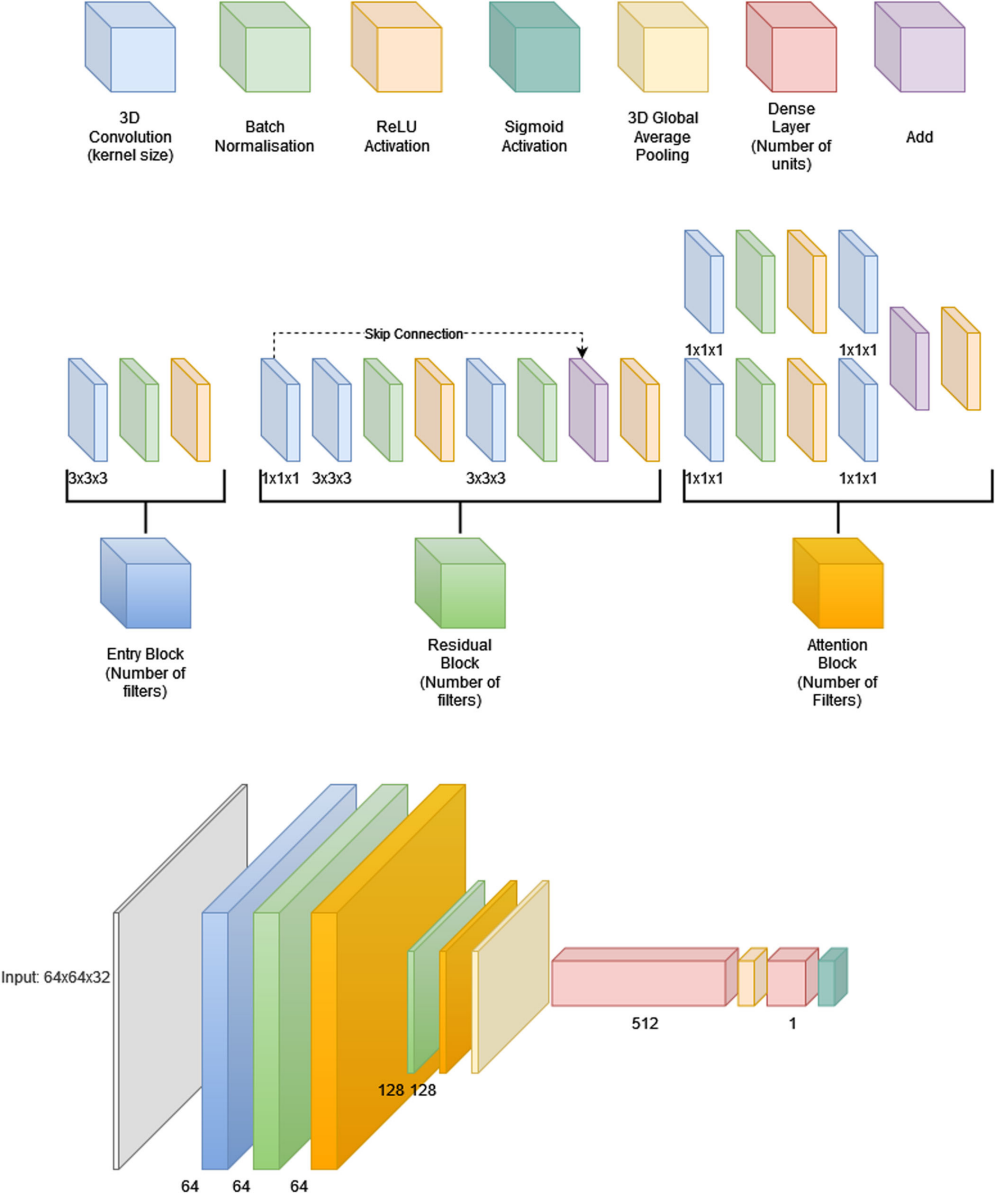
The 3D CNN model architecture. In the diagram, the flow of data between the blocks is from left to right.

Three 3D CNN models utilising residual connections and an attention mechanism were built with PET, MRI, and PET/MRI images using Python version 3.10.11 and Tensorflows Keras version 2.10.1 (Martín Abadi et al., 2015). The model consists of an entry block utilising a 3D convolutional layer together with batch normalisation and ReLU activation. After the entry block, the data is passed through a residual and an attention block with 64 filters each. The attention block refines features by passing them through two parallel 1×1×1 convolutions that learn different channel‐wise transformations. The outputs are then merged and reactivated. This is followed by a second pass through a residual and an attention block with 128 filters each. After this, a global average pooling (GAP) layer together with a dense layer of 512 nodes and a sigmoid activation was used to produce the final binary classification. The model architecture is depicted in Figure 1. GAP was used to produce fixed‐length feature vectors independent of the spatial dimensions of the input patch, allowing the model to process volumes with varying depths without modifying the classifier architecture, reducing trainable parameters and mitigating overfitting. Despite enabling size invariance with GAP, inference was conducted using a sliding window to address practical issues. First, memory constraints of the GPU used for training prevent from processing the entire volume at once. Second, relevant pathological findings are often localised to narrow regions and whole‐volume averaging could dilute these signals. Third, aggregating predictions from overlapping sub‐volumes may improve robustness with reduced sensitivity to local noise or artifacts.

The Adam optimiser was used with an initial learning rate of 0.0001 and an exponential decay rate of 0.96 with 100 000 steps. Focal binary cross‐entropy was used as the loss function and area under the receiver operating characteristic (ROC) curve (AUC) as the metric for the loss function during training.

### 3.4 Quantitative model performance evaluation

To ensure robust evaluation, a stratified fivefold cross‐validation was used when training and validating the model. The classification performance of the model for each fold was evaluated in terms of the sensitivity, specificity, and accuracy in the validation set. In addition, ROC curves were drawn, and AUC values were calculated for each validation set of the fivefolds. The optimal threshold for the binary classification was determined using the Equitability measure (Rainio et al., 2024). To account for overfitting, early stopping was used, and the weights of the epoch with the highest validation scores were restored. An ensemble of the cross‐validation models was then evaluated on a separate test set using the same metrics as in the validation procedure. The ensemble consisted of all five models trained during the cross‐validation for a given image modality. This process was conducted on PET, MRI, and PET/MRI data to assess differences in performance between the imaging modalities. Inference on the test set was conducted using a sliding window with a window size of 32 and a stride of 16. Overlapping windows mitigate the risk of missing a tumour visible in only a single window. Four different methods of aggregating the sliding window results to obtain a final classification were evaluated: average, majority vote, median, and weighted average. The majority voting produces a positive classification if the majority of the predicted sliding windows are classified as positive; otherwise, a negative classification is provided. The weighted average was implemented in a way that linearly reduces the impact of the slices closer to the top of the head based on the number of evaluated sliding windows. This was done to mimic the clinical understanding where HNC is typically not seen above the nasopharynx level, yet the images contain slices above it with high metabolic activity from the brain. We chose mean/majority‐type aggregation to stabilise predictions against spurious single‐window false positives, while the overlap preserves sensitivity.

Sensitivity for the classification was calculated as follows:

$$\mathrm{Sensitivity}=\;\frac{\mathrm{TP}}{\mathrm{TP}+\mathrm{FN}}.$$

Specificity for the classification was calculated as follows:

$$\mathrm{Specificity}=\;\frac{\mathrm{TN}}{\mathrm{TN}+\mathrm{FP}}.$$

Accuracy for the classification was calculated as follows:

$$\mathrm{Accuracy}=\;\frac{\mathrm{TP}+\mathrm{TN}}{\mathrm{TP}+\mathrm{TN}+\mathrm{FP}+\mathrm{FN}}.$$

### 3.5 Model interpretability with Gradient‐Weighted Class Activation Mapping (Grad‐CAM)

With the aim of reducing the inherent black box—nature of deep CNNs, a Grad‐CAM was built to visualise the intermediate network gradients, allowing us to better understand the focal areas in the model′s decision‐making process. This approach helps identify the regions in the input image that the model focuses on when making decisions.

### 3.6 Comparison with a radiologist

The test set was also evaluated by an experienced head and neck radiologist who had no prior knowledge of the reference standard (histopathological proof) but knew the location of the primary tumour, except the one unknown primary case in the test set. The radiologist examined the PET/MRI images and gave a binary verdict indicating the absence or presence of any residual disease activity in the primary location or new disease activity at a secondary location.

## 4 RESULTS

### 4.1 Cross‐validation

**FIGURE 2:**
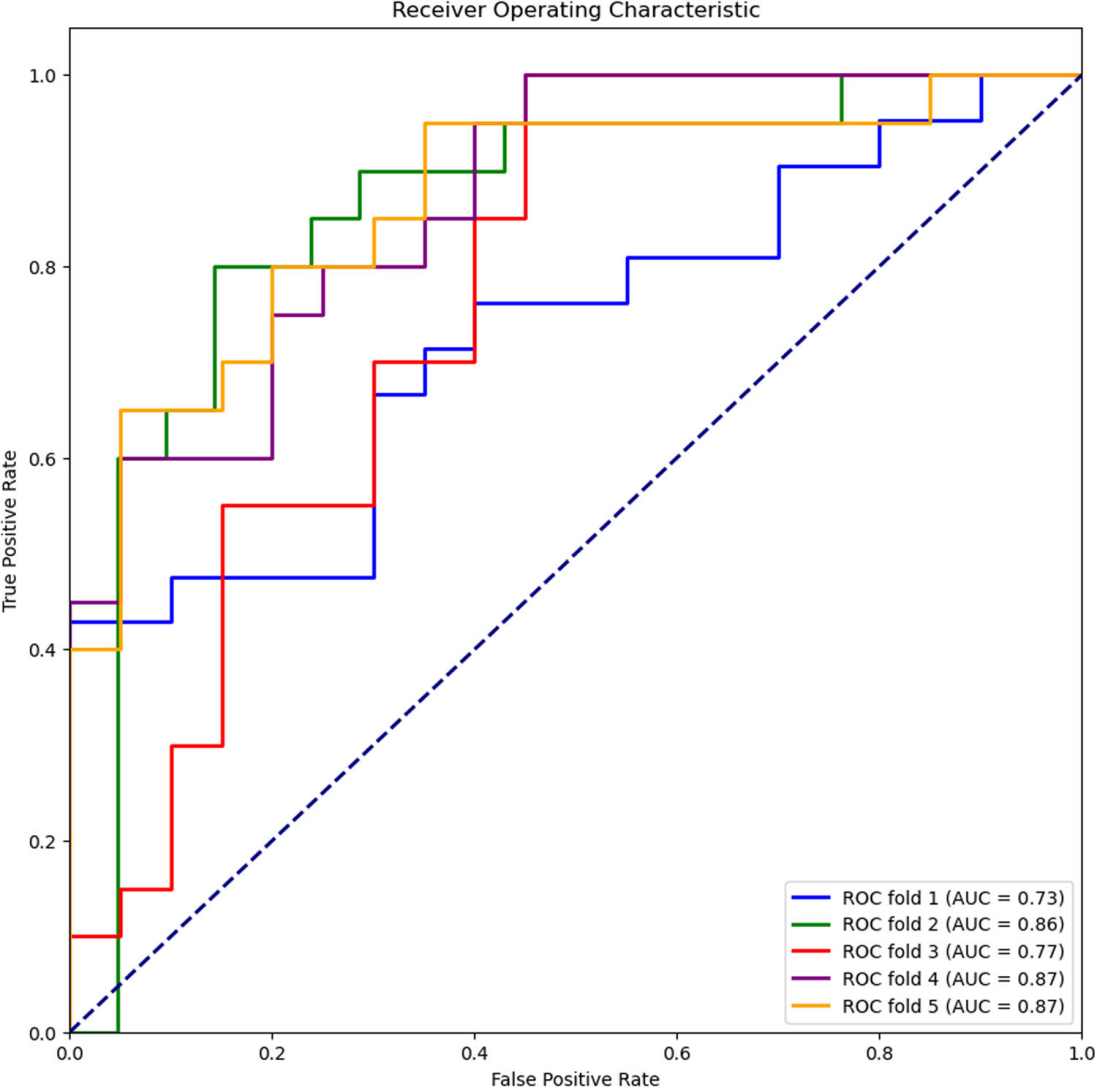
ROC curves and AUC values of the fivefold cross‐validation procedure for the PET data.

In the cross‐validation, some overfitting and volatility between epochs were observed during training for all imaging modalities, which is to be expected with these sample sizes and heterogeneity within the samples. Between the imaging modalities, PET and PET/MRI achieved similar results, whereas MRI scored lower (Table 5). Across the 5 validation folds, the PET‐based model achieved a mean accuracy, sensitivity, specificity, and AUC of 0.75, 0.72, 0.77, and 0.82, respectively. For the MRI‐based models, these values were 0.66, 0.63, 0.68, 0.73, and for the PET/MRI‐based model, they were 0.72, 0.69, 0.74, and 0.80. The AUCs achieved during the fivefold cross‐validation by the PET‐based models are depicted in Figure 2. The true negatives, false positives, false negatives, and true positives for all models and all validation folds are listed in Table 6.

**TABLE 5:** Mean and median performance across the training folds during the fivefold cross‐validation procedure.

Data	Method	Accuracy	Sensitivity	Specificity	AUC
PET					
	Mean	**0.75**	**0.72**	**0.77**	**0.82**
	Median	**0.78**	**0.75**	**0.80**	**0.86**
MRII					
	Mean	0.66	0.63	0.68	0.73
	Median	0.68	0.65	0.70	0.74
PET/MRI					
	Mean	0.72	0.69	0.74	0.80
	Median	0.73	0.70	0.75	0.81

*Note*: Highest values are bolded.

**TABLE 6:** Classification results for all validations folds for each model.

Modality	Validation fold	True negatives	False positives	False negatives	True positives
PET	1	14	6	7	14
	2	18	3	4	16
	3	14	6	7	13
	4	16	4	5	15
	5	16	4	5	15
MRI	1	15	5	6	15
	2	13	8	9	11
	3	15	5	6	14
	4	12	8	9	11
	5	14	6	7	13
PET/MRI	1	15	5	6	15
	2	15	6	7	13
	3	13	7	8	12
	4	17	3	4	16
	5	15	5	6	14

### 4.2 Test set

After the fivefold cross‐validation procedure, an ensemble of the models for each imaging modality was used to classify the independent test set described previously. In terms of accuracy, averaging over the sliding windows or taking the median proved to be the best option for PET, MRI and PET/MRI‐based models, with these models achieving accuracies of 0.90, 0.75 and 0.60, respectively. For the PET/MRI and PET models, the highest sensitivities of 0.70 and 1.0 were obtained using average, median, or weighted average. For the MRI‐based model, the highest sensitivity was achieved with weighted average. For PET and MRI‐based models, the highest specificities of 0.80 and 0.90, respectively, were observed using averaging, majority voting and median. For the PET/MRI‐based model, the highest specificity of 0.50 was seen regardless of the method. For AUC, the weighted average performed best for PET scoring 0.94. For PET/MRI, the highest AUC was seen when taking the median of the sliding windows. For MRI, the best AUC of 0.79 was achieved with averaging. A summary of these different methods of aggregating the sliding window inference for each model is depicted in Table 7. Due to the implementation of majority voting, no AUC scores were obtained with this method. Moreover, the test set was evaluated by an experienced head and neck radiologist, who was not aware of any prior patient information other than them being HNC patients. The radiologist identified all cases with perfect accuracy.

**TABLE 7:** Sliding window inference on the test set and comparison with a radiologist.

Data	Method	Accuracy	Sensitivity	Specificity	AUC
PET/MRI					
	Average	0.60	0.70	0.50	0.72
	Majority vote	0.55	0.60	0.50	NA
	Median	0.60	0.70	0.50	0.74
	Weighted average	0.60	0.70	0.50	0.73
PET					
	Average	**0.90**	**1.00**	**0.80**	0.92
	Majority vote	0.70	0.60	0.80	NA
	Median	**0.90**	**1.00**	**0.80**	0.92
	Weighted average	0.75	1.00	0.50	**0.94**
MRI					
	Average	0.75	0.60	0.90	0.79
	Majority vote	0.60	0.30	0.90	NA
	Median	0.75	0.60	0.90	0.77
	Weighted average	0.70	0.70	0.70	0.78
Radiologist		**1.00**	**1.00**	**1.00**	NA

*Note*: Highest values are bolded.

### 4.3 Grad‐CAM

**FIGURE 3:**
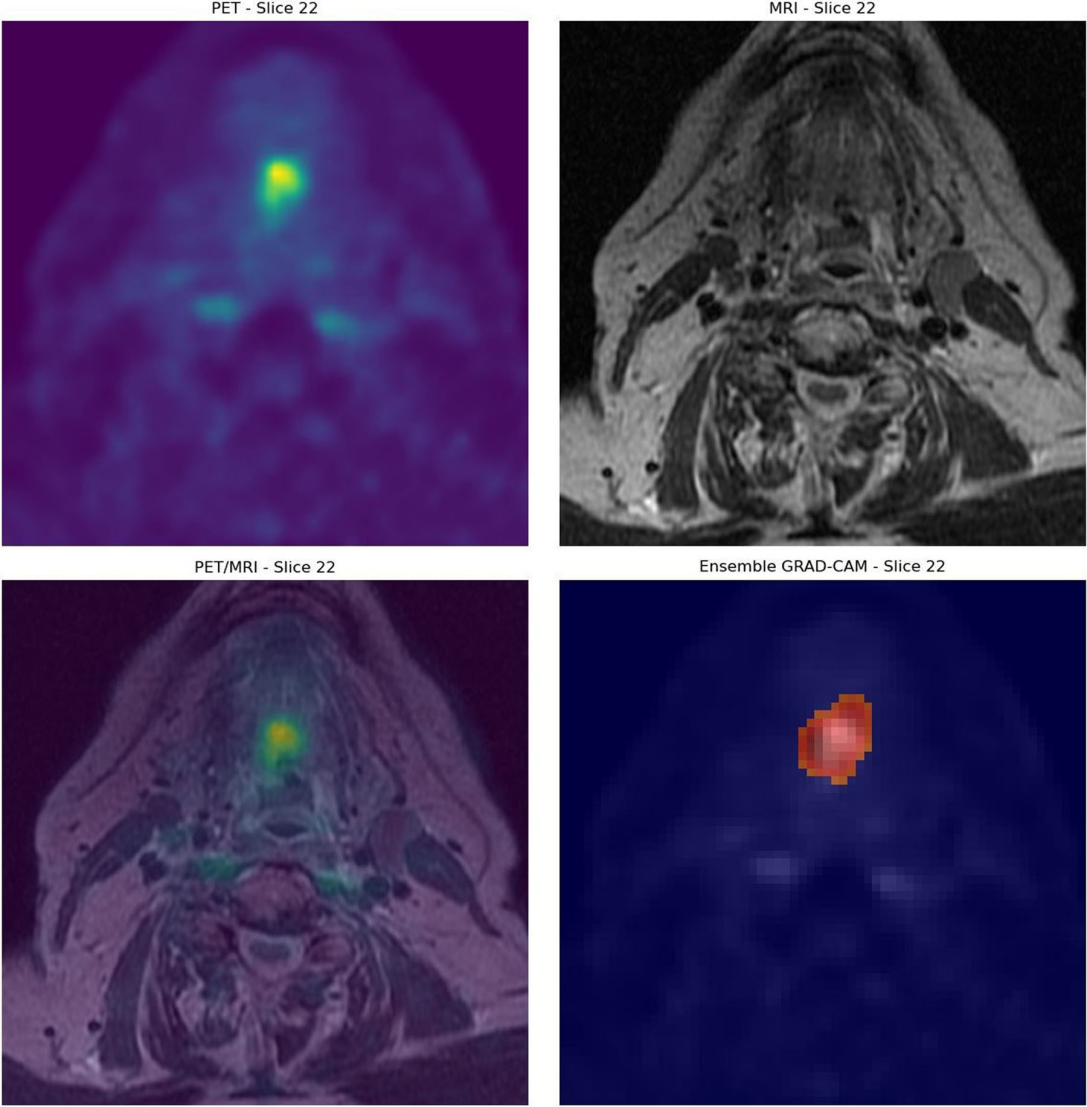
Example of Grad‐CAM on a correctly classified patient with significant residual SCC of the tongue. Grad‐CAM, Gradient‐Weighted Class Activation Mapping.

**FIGURE 4:**
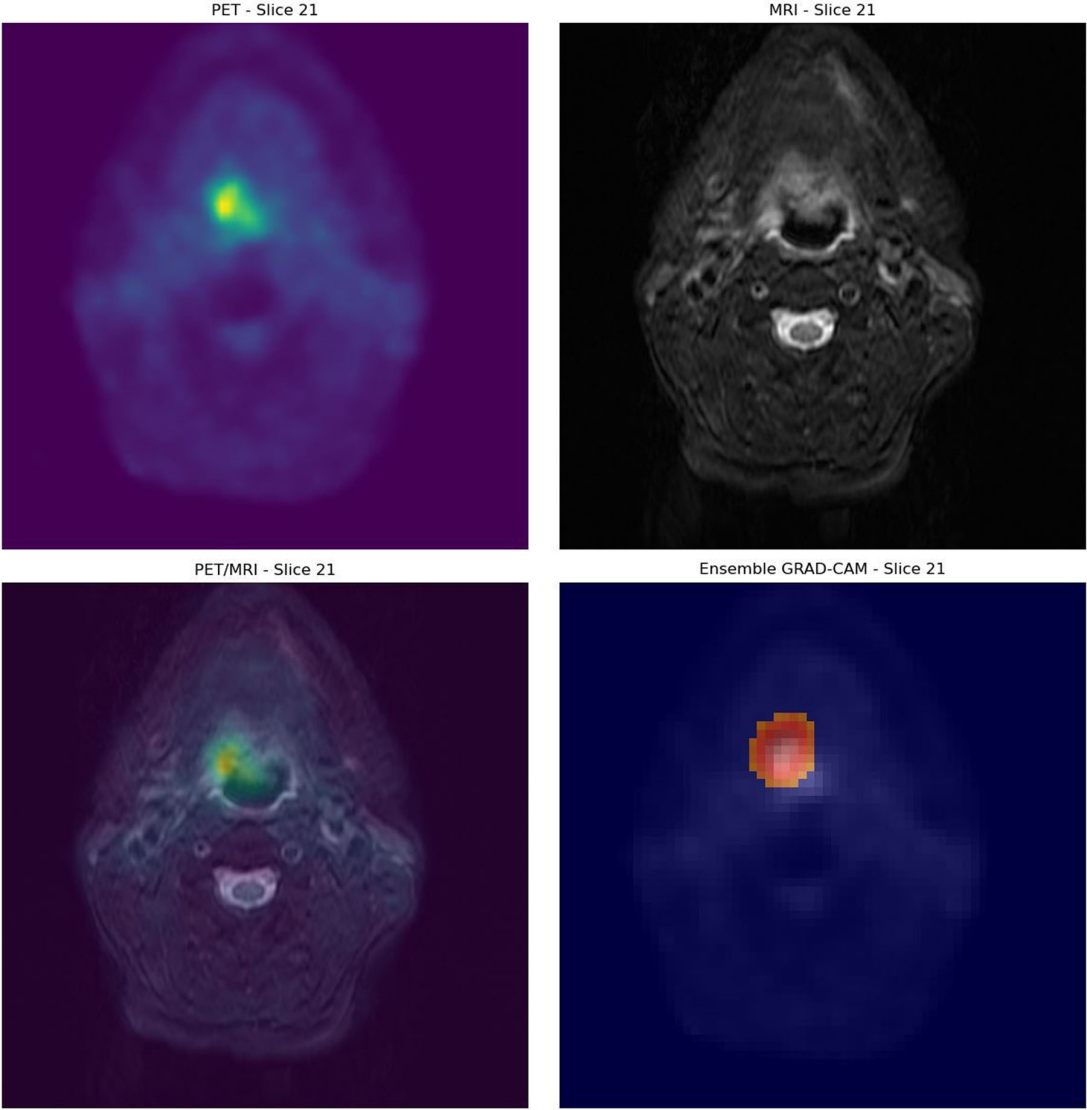
Example of Grad‐CAM on a correctly classified patient with significant residual SCC of the oropharynx. Grad‐CAM, Gradient‐Weighted Class Activation Mapping.

The highest‐performing PET‐based model was then evaluated in terms of interpretability using Grad‐CAM. Grad‐CAM suggests that the positive predictions are made correctly made based on areas of high malignant metabolic activity. An example of this is depicted in Figure 3, where a patient with significant residual disease (SCC of the base of the tongue) was classified correctly as positive. Similar example of a patient that was correctly classified as positive is shown in Figure 4. This patient had a SCC of the oropharynx.

**FIGURE 5:**
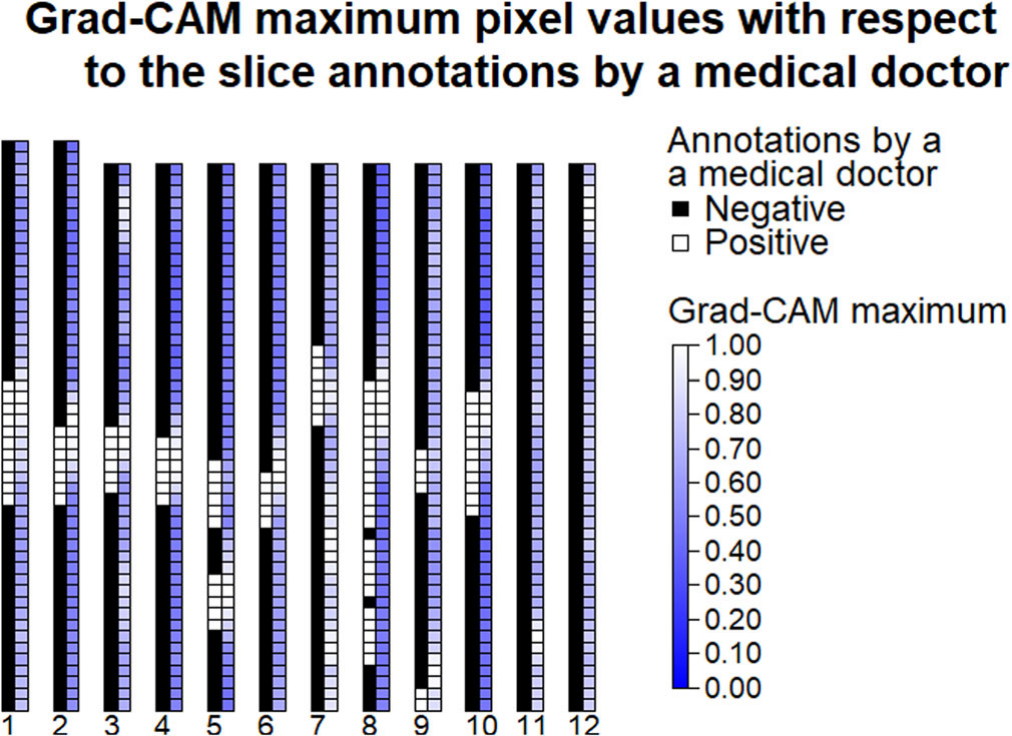
Correlation between the maximum pixel value per slice in Gradient‐Weighted Class Activation Mapping (Grad‐CAM) and binary ground truth values for image slices in patients classified as positive. *X*‐axis indices 1–10 depict the 10 patients of the test set that were classified correctly as positive, and indices 11–12 depict those that were falsely classified as positive. Pixel intensity of Grad‐CAM correlates with the depth‐axis location of the malignant tissue in the true positive patients indicating that the true positive classifications are based on real findings. However, because the Grad‐CAM is based on the PET‐model, malignant areas without significant metabolic activity, such as necrotic lymph nodes, might get overlooked, which explains the transaxial slices, where there is cancer, yet only low or moderate Grad‐CAM intensity.

To demonstrate the connection between the model′s observations and the final classification results, the correlation between the maximum pixel value of Grad‐CAM per image slice and image slices containing cancer was explored. Results of this comparison for all patients classified as positive (10 true positives and 2 false positives) are illustrated in Figure 5. In this graph, a single box in a column represents one transaxial PET slice. The left column represents the binary ground truth per transaxial slice, where black is negative and white is positive, that is, is there cancer present on this transaxial slice in the original images. The right column depicts the maximum intensity of a single pixel for a given transaxial slice of the Grad‐CAM heatmap on a scale from 0 to 1. For the patients that were correctly classified as positive (1–10), overlap in Grad‐CAM activity and positive slices was observed. Unsurprisingly, no overlap was observed for the false positives, as all image slices are negative.

### 4.4 PET model failures on the test set

The best‐performing model was the PET‐based model utilising averaging (and median) for sliding window aggregation. This model achieved a sensitivity of 100% and a specificity of 80% on the test set, meaning that the model predicted 2 false positives. The raw predictions for these cases were 0.92 and 0.88, while the decision threshold for the ensemble model was 0.87 (mean of the 5 thresholds obtained during training).

**FIGURE 6:**
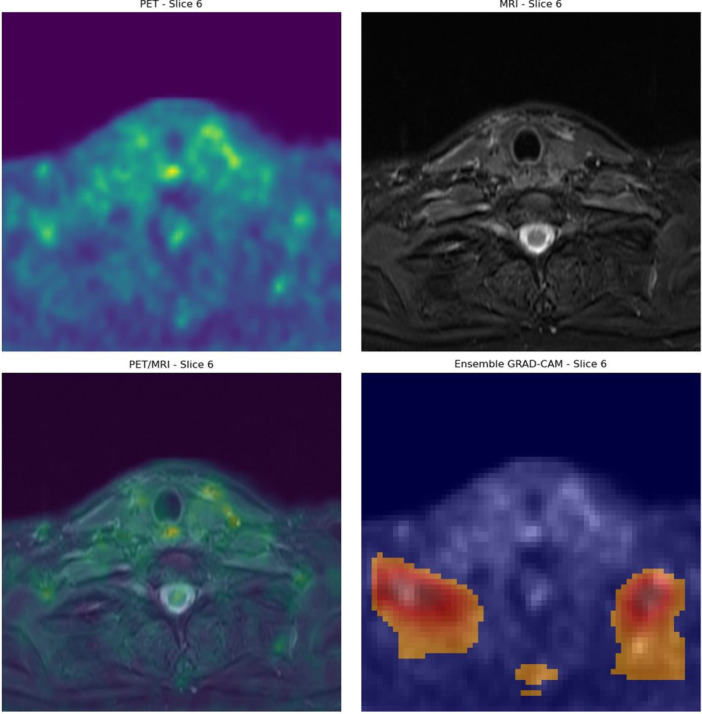
Grad‐CAM of the first false positive classification. Grad‐CAM, Gradient‐Weighted Class Activation Mapping.

The first one of these was a patient who had successfully been treated for SCC of the left tonsil. The PET/MRI shows abnormal metabolic activity in the area, which, however, was not malignant. While correctly classifying this patient as negative, the radiologist also noted that nonspecific activity was seen outside the primary tumour area. However, examining the Grad‐CAM, no particular regions of interest were found in this area. Instead, the model seems to focus most on the shoulder muscles as seen in Figure 6.

**FIGURE 7:**
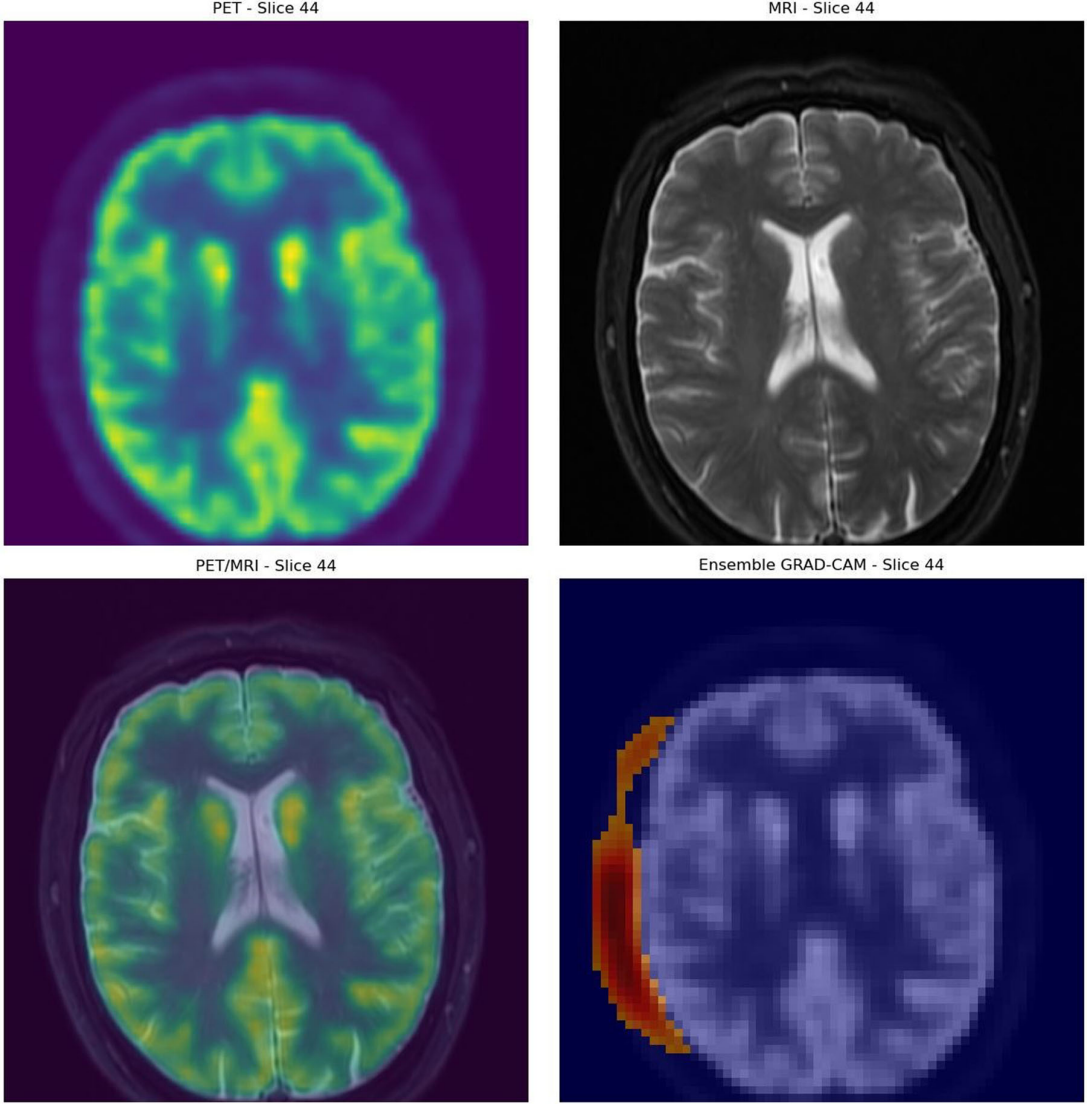
Grad‐CAM of the second false positive classification. Grad‐CAM, Gradient‐Weighted Class Activation Mapping.

The second false positive was a patient treated for epithelial cancer of the right sinus. This patient′s PET/MRI had prominent but diffuse metabolic activity in the sublingual salivary gland, which was benign. However, Grad‐CAM showed that the model′s attention focused most intensely on a section of the skull as depicted in Figure 7.

## 5 DISCUSSION

The achieved classification results are promising, especially for the PET‐based model. The results of the PET model align well with our previous work on the subject (Hellström et al., 2023). Similarly, the poorer performance of the MRI‐based model is not surprising considering our prior experience in comparing PET, MRI, and PET/MRI in 2D segmentation of HNC (Liedes et al., 2023). Combining PET with MRI surprisingly resulted in worse classification results on the test set than either of the modalities alone. Similar phenomenon was not observed during cross‐validation, where PET/MRI based models were superior to MRI‐based. However, even during cross‐validation the PET‐based models outperformed the PET/MRI models slightly. This could be related to the relatively limited sample size and heterogeneity within the data, which could interfere with the model′s ability to pick up patterns when the two input channels are used together. This type of behaviour might be especially prevalent in instances where the majority of identifiable tumours have high metabolic activity, as is the case in our data. This is in contrast with situations where the tumours exhibit less or no metabolic activity, for example due to necrosis. We suspect this issue could be fixed by acquiring more such data. Furthermore, it is likely that the models have an easier time learning patterns of high metabolic activity due to the way the images are normalised before training. Similarly, due to the choice of loss function, the models are driven to emphasise pathological regions, since tumour voxels are naturally less prevalent than normal tissue. As a result, the PET‐based models may favour pattern intensity over anatomical features that are seen in MRI images. Moreover, in a recent meta‐analysis no statistically significant differences were observed in terms of sensitivity or specificity when PET/MRI was compared to MRI in HNSCC diagnosis, suggesting that fusing the modalities does not always guarantee improvement in clinical accuracy (Al‐Ibraheem et al., 2024).

Overfitting was observed during the training and cross‐validation process. However, the PET‐based ensemble model achieved better accuracy, sensitivity, and AUC on the test set than the average of the same metrics during cross‐validation, demonstrating the generalisation abilities of this model in particular. In addition to our previous work on the subject, we have shown here that analysing whole 3D PET/MRI stacks with variable depths using a sliding window approach is feasible and accurate in the binary classification of HNC patients. This method also compares favourably in terms of efficiency, enabling direct analysis of entire 3D images, unlike 2D methods that would require aggregating the classification of each individual image slice to produce the same patient‐wise classification result, which is what ultimately carries the clinical value. Moreover, this study adds to our previous work by examining Grad‐CAM as a useful interpretability measure in DL assisted PET/MRI analysis for HNC.

To the best of our knowledge, comparable 3D cancer classification of HNC patients from PET‐MRI images has not been carried out previously. However, similar work describing tumour classification of other regions and/or other imaging modalities has been published. Although technically not a 3D method, Kawauchi et al. described a deep learning based method for classifying whole body ^18^F‐FDG PET scans as benign, malignant or equivocal utilising maximum intensity projection images as data (*N* = 3485) (Kawauchi et al., 2020). They conducted a region‐based analysis where their model predicted correctly with a probability of 97.3% for the head and neck area. Liu et al. explored the use of 3D CNNs in distinguishing benign inverted papillomas from those that have undergone malignant transformation in MRIs (*N* = 90) (Liu et al., 2022). Their best model achieved an accuracy of 77.9% and an AUC of 0.80. Similarly, Wong et al. achieved an accuracy of 91.5% and AUC of 0.96 in classifying nasopharyngeal carcinoma from benign hyperplasia on MRIs (*N* = 412) using a 3D CNN (Wong et al., 2021). Considering the purposeful heterogeneity of our data, our results are in line compared with the forementioned studies.

The trained models were evaluated against the gold standard of histological confirmation for positive cases and absence of disease at follow‐up for negative cases. For comparison, an experienced head and neck radiologist also reviewed the images. In this data set, the radiologist's classifications were in complete agreement with the gold standard, thereby achieving perfect accuracy and clearly outperforming the models. Since clinical practice suggests that the radiologists' performance is not perfect, a larger sample might have revealed more human reader errors. In addition, a more fine‐grained response score (e.g., a five‐point scale instead of a dichotomous outcome) might have better illustrated the uncertainties encountered in the clinical workflow. Moreover, the PET/MRI‐based model was unable to gain any additional benefit from the MRI data and performed worse than the PET‐based model. Addressing this issue with more robust data sets could help the models benefit from the combined PET/MRI data in the same way a radiologist does.

With the aim of increasing the interpretability of our deep learning models, we implemented Grad‐CAM. Firstly, we found that the pixel intensity of Grad‐CAM seems to correlate with the depth‐axis location of the malignant tissue in the positive patients. Thus, indicating that the true positive classifications are based on real findings. However, because the Grad‐CAM is based on the PET‐model, malignant areas without significant metabolic activity, such as necrotic lymph nodes, can get overlooked, which explains the transaxial slices in Figure 5, where there is cancer, yet only low or moderate Grad‐CAM intensity. It should also be noted that comparing the maximum value of a single pixel per slice with the ground truth value of the entire slice is a simple proxy to help visualise the depth‐wise attention distribution of the model. This method does not consider, for example, the number of high‐intensity pixels (i.e., the size of the region of interest). Moreover, the model might also make some interpretations based on the presence of normal anatomical structures, which, however, is not reflected in Figure 5, where only the correlation between model attention and cancerous transaxial slices is depicted. Examining the Grad‐CAMs from the two false positive classification cases, there does not seem to be any clear reason why the model's peak interest would be on the sections shown in Figures 6 and 7. From a radiologist's perspective, this can be a beneficial finding, as it makes identifying false positives easier when these systems are applied in practice.

The PET model performed with perfect sensitivity and a specificity of 80%, which has positive clinical implications as this system could be utilised as an efficient pre‐screening step in HNC PET/MRI interpretation. In terms of efficiency, the deep learning models obtain a classification for a single patient in under a second, significantly outperforming human observers. Coupled with mechanisms that enhance interpretability, such as Grad‐CAM, which allow easy identification of false positives, these systems could provide significant benefits to the clinical workflow. Further benefit of using the PET‐based model is its compatibility with both MRI and CT.

Some limitations to our study need to be addressed. Firstly, the sample sizes for our training and test sets were limited when considering the difficulty of this problem. In addition, the samples are from the same centre. HNCs are a diverse group of tumours with greatly varying anatomic locations. We chose to include all types of cancer occurring in the head and neck area to better mimic potential real‐life applications of these algorithms. However, this comes with obvious challenges as SCCs of the oral cavity, pharynx, and larynx were over‐represented in the data, and other types of cancer had very few samples of each. Secondly, both the training and test sets were equally distributed in terms of negative and positive cases, while in clinical practice, negative cases are more common. In addition, the preprocessing applied to the data before training and testing includes significant downsizing of the images to make training possible with limited local computing resources (Nvidia GeForce RTX 3070). This process reduces the image quality and can therefore influence the classification accuracy, especially with small tumours requiring high fidelity. This issue could be mitigated in future studies by increasing compute in a cloud environment that meets the high security requirements of this sensitive data.

Moreover, the current rate of false‐positive classifications is too high for reliable use in clinical practice. While Grad‐CAM improves interpretability and provides some insight into model decisions, it only partially addresses the issue. Long‐term improvements will require more capable models, which depend on larger, more diverse, and robust training datasets. A key method for obtaining more data in future work may be more advanced data augmentation schemes and/or generative networks. Addressing these limitations will enable the integration of such systems into clinical practice, where they can serve effectively as pre‐screening tools. These systems would provide clinicians with initial classifications, which could then be reviewed and interpreted using Grad‐CAM. Once a positive case is confirmed, it could undergo further analysis using deep learning‐based segmentation, allowing clinicians to refine or adjust the diagnosis as needed. These types of workflows would enhance diagnostic precision and efficiency while keeping clinicians in control of critical decision points.

## 6 CONCLUSIONS

The PET‐based deep learning model achieved perfect sensitivity on the test set, showing promise as an effective pre‐screening tool for HNC diagnosis. In addition, increasing model interpretability through tools such as Grad‐CAM can provide valuable insights into model decisions, while increasing trust among clinicians. However, specificity requires improvement to reduce false‐positive rates and to improve the clinical utility. We anticipate that advancements in data set quality and size, optimisation of pre‐processing protocols, and enhancements to model architecture and interpretability will allow us to benefit from PET/MRI fusion imaging fully, rather than relying solely on PET‐based models.

## CONFLICT OF INTEREST STATEMENT

The authors declare no conflicts of interest.

## Data Availability

The data that support the findings of this study are available on request from the corresponding author. The data are not publicly available due to privacy or ethical restrictions.
